# A rare case of failed healing in previously burned skin after a secondary burns

**DOI:** 10.1186/s41038-017-0099-3

**Published:** 2017-12-04

**Authors:** Stephen J. Goldie, Shaun Parsons, Hana Menezes, Andrew Ives, Heather Cleland

**Affiliations:** 1 Victorian Adult Burns Service, Alfred Hospital, Melbourne, Australia; 2 Department of Surgery, Monash University, Melbourne, Australia

**Keywords:** Previous burns, Burn scar, Non-healing

## Abstract

**Background:**

Patients presenting with large surface area burns are common in our practice; however, patients with a secondary large burn on pre-existing burn scars and grafts are rare and not reported.

**Case presentation:**

We report on an unusual case of a patient sustaining a secondary large burn to areas previously injured by a burn from a different mechanism. We discuss the potential implications when managing a case like this and suggest potential biological reasons why the skin may behave differently. Our patient was a 33-year-old man who presented with a 5% TBSA burn on skin scarred by a previous 40% total body surface area (TBSA) burn and skin grafts. Initially assessed as superficial partial thickness in depth, the wounds were treated conservatively with dressings; however, they failed to heal and became infected requiring surgical management.

**Conclusions:**

Burns sustained in areas of previous burn scars and grafts may behave differently to normal patterns of healing, requiring more aggressive management and surgical intervention at an early stage.

## Background

In our practice at the Victorian Adult Burns Service (VABS), we admit on average 400 burns patients per year and aim to have their wounds healed within a strict 3-week time frame in order to reduce the long-term burn sequelae of scarring and contractures. Burn depth is assessed as superficial, partial thickness or full thickness based largely on clinical assessment. Laser Doppler scanning is utilised as an adjunct assessment in partial thickness burns which are borderline superficial partial thickness and likely to heal or deep partial thickness that will likely need surgical intervention. In our practice, superficial partial thickness burns covering an area greater than 5% total body surface area (TBSA) and presenting within 48 h post burn may be considered for surgical scrubbing of the wounds and application of Biobrane. Superficial partial thickness burns covering less than 5% TBSA may be debrided and dressed as required. On review in clinic, these smaller, more superficial burns invariably heal within the 3-week time frame. Here, we report the case of a patient who did not conform to our usual treatment algorithm due to a previous burn injury.

## Case presentation

A 33-year-old male was brought to the emergency department by ambulance having been exposed to an explosive flash burn from a nitrous compressor while welding. The patient sustained burns to the face, neck, hands, and arms. His immediate first aid was to jump into a swimming pool of cold water. His burns were clinically assessed as 5% TBSA and mainly of superficial partial thickness. The epidermal layer had blistered and been removed, but the underlying dermis remained sensate and with a brisk capillary refill. The patient was known to the VABS as he had sustained a 40% TBSA burn 12 years previously, following a fire while cooking with hot oil. Remarkably, all of the flash burns from the welding accident had affected previously grafted or scarred skin. The patient was admitted for 48 h for analgesia and to establish a dressing plan and face care protocol on the ward. Once comfortable, he was discharged home to have dressings in the community and for clinic follow-up; within an expectation, his wounds would heal within the 3-week target.

**Fig. 1:**
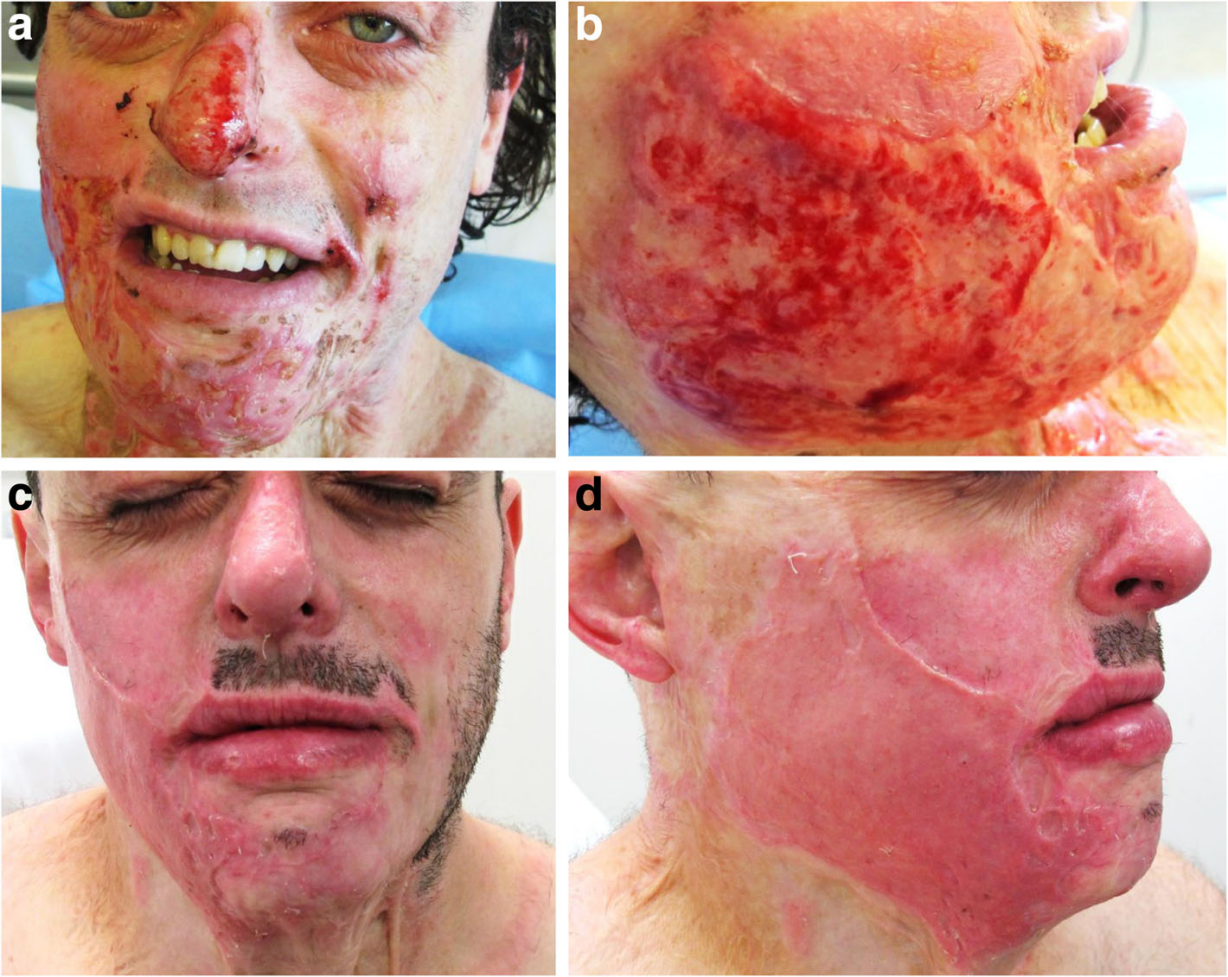
**a**, **b** Facial burns on previous burn scars on readmission, prior to grafting. The wounds were cleaned on the ward to remove a thick layer of slough. **c** Sheet grafting of the scalp skin to nose and **d** right cheek, 2 weeks post grafting

**Fig. 2:**
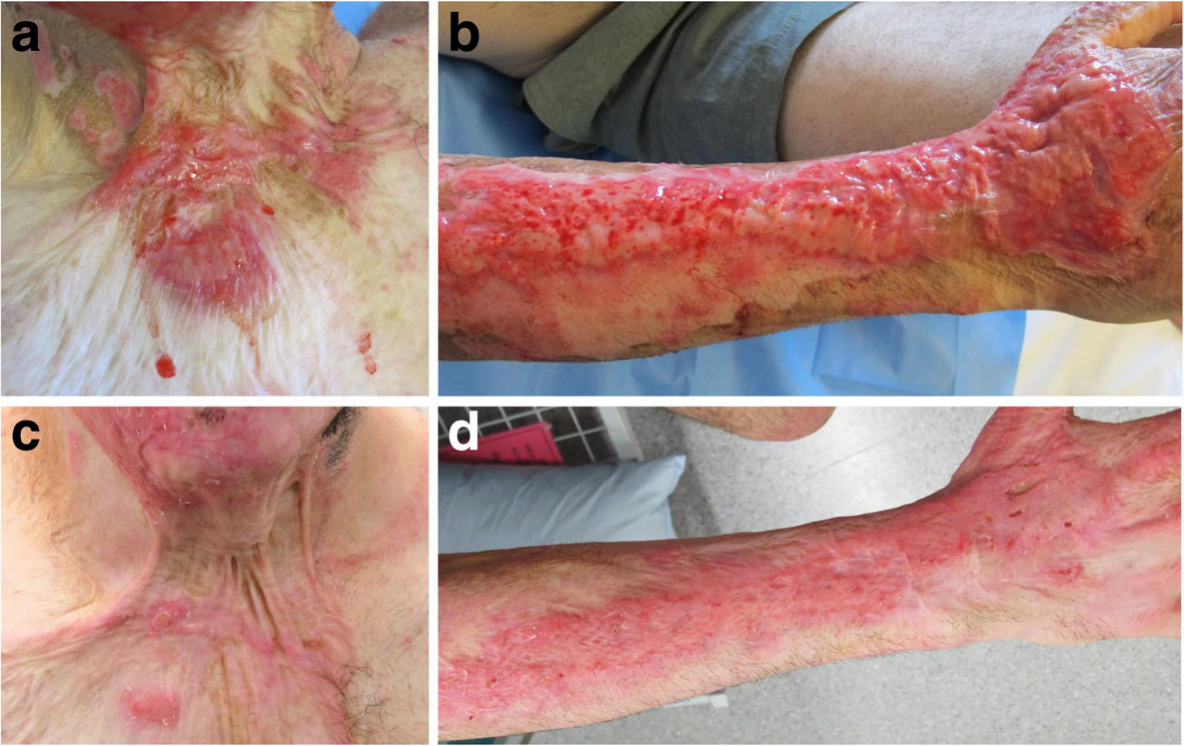
**a** Burns to the neck and **b** the dorsum of the right arm and hand, on previous burn scars on readmission, prior to grafting. The wounds were cleaned on the ward to remove a thick layer of slough. **c** Meshed grafting to the neck and **d** right forearm and hand, 2 weeks post grafting

Five days later, the patient re-presented to the emergency department feeling feverish and complaining of general malaise. On examination, he had sloughy, infected burn wounds on the face, neck, hands, and arms. He was swabbed for microbiology, then commenced on intravenous antibiotics. Despite the wounds having been previously assessed as superficial partial thickness, it was apparent that the burns had failed to improve and would not heal without surgical intervention (see Figs. 1a, b and 2a, b). The burns were subsequently debrided in theatre with the Versajet II (hydrosurgery system), before being autografted. Split thickness thigh skin was used to sheet graft the dorsum of the hands with Artiss (fibrin sealant) to improve adhesion. Scalp skin was harvested to better match the skin of the face and neck. In addition due to the large amount of skin previously harvested from the patient’s thighs in his previous burn surgery, it was thought that further use of these areas for grafting of the face would give suboptimal results. Scalp skin was inset on the face as sheet grafting with Artiss. Thigh skin meshed 2:1 was used to reconstruct the less cosmetically sensitive areas on the arms. Grafts were checked at 3 days and found to be intact and adhering well (see Figs. 1c, d and 2c, d). The patient was discharged home satisfied that his new grafts had improved the cosmesis of his previously scarred facial burns.

**Fig. 3:**
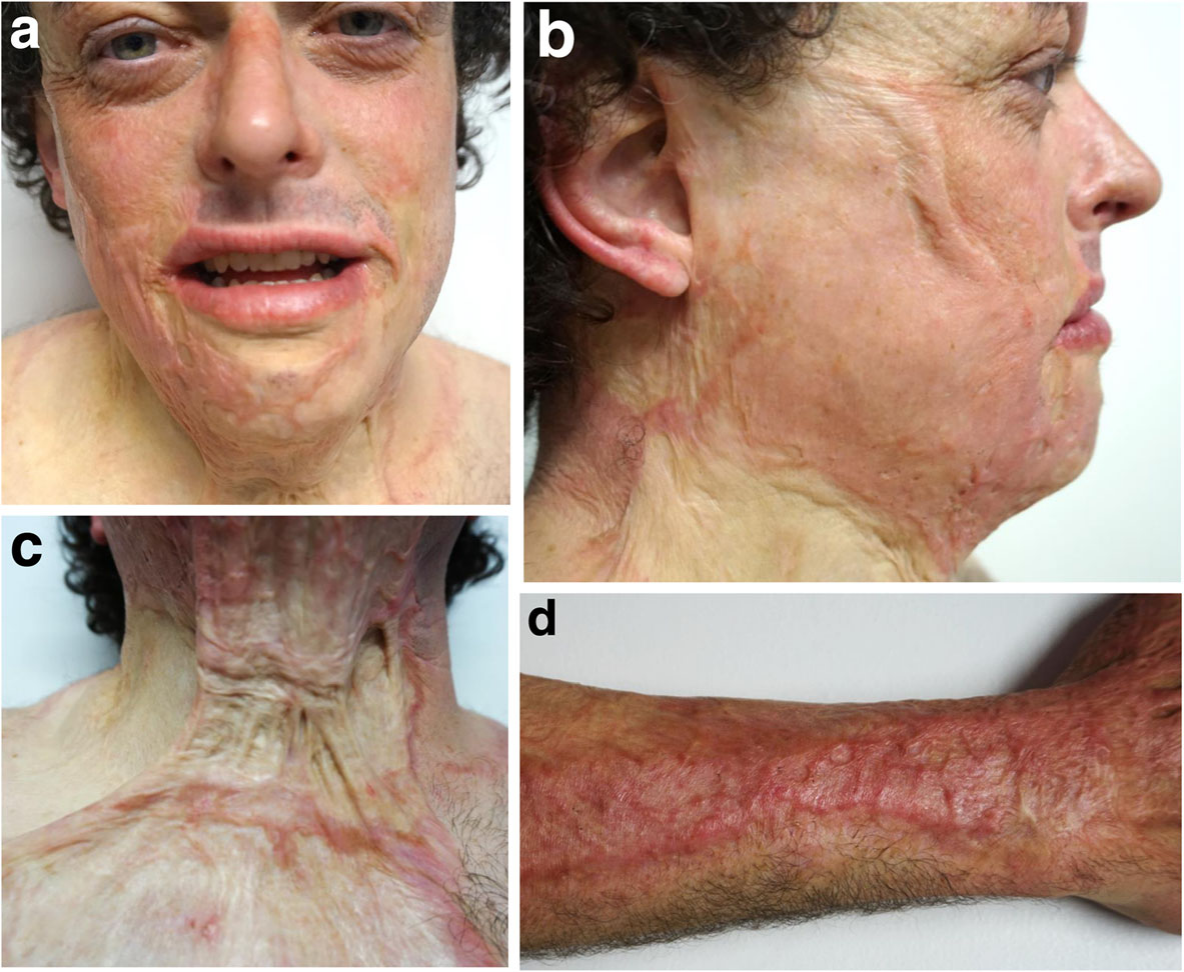
Long-term follow-up (7 months) of grafted burn wounds to **a**, **b** the face, **c** neck, and **d** right forearm

Seven months following surgery, the patient continues to attend clinic for long-term follow-up of his wounds. As is the usual practice in our department, he is undergoing treatment with pressure garments and silicone in order to keep scarring to a minimum and prevent contractures (see Fig. 3).

## Discussion

This case of a 33-year-old man who sustained flash burns onto previously burned and grafted skin highlights the issues of poor long-term durability and healing ability in skin grafted or scarred skin. The risk of burns from welding has been long established [1, 2]; however, few reports exist of how the wound behaves when a patient sustains burns on previously scarred skin. The human epidermis is multilayered with a stem cell niche not only amongst the basal cells at the basement membrane, but also within the specialised skin appendages, i.e. the hair follicles and sebaceous glands which project into the underlying dermis [3]. Burn wounds that have been reconstructed with split thickness skin grafts may not include all of these particular stem cell niches and lose the normal interaction and communication with the underlying dermis. Signalling between the epidermis and dermis, through the basement membrane, is crucial during development [4] and is likely to be equally important in regenerating skin during wound healing. Without these cell-cell interactions, the skin may heal but does not regenerate a complete functional facsimile of the unburnt skin. The skin which has been depleted of stem cells by injury or age is less able to respond to the demands of switching from homeostatic maintenance of the epidermis to the rapid proliferation required during wound healing [5, 6].

## Conclusions

Our experience in this case and reading of current skin biology literature would lead us to re-evaluate our practice and modify our future management of subsequent similar cases. In the context of thermal injuries onto previously burned skin, we would recommend more cautious interpretation of clinical findings and to assume that the wound will not behave as would be expected of normal skin. We would recommend more aggressive debridement of these wounds and early grafting as appropriate. As with our case, this may inadvertently benefit the patient in improving the aesthetic quality of the scarred skin.
